# Supplementary material for: How to entrain a selected neuronal rhythm but not others: open-loop dithered brain stimulation for selective entrainment
Source: J Neural Eng. Author manuscript; Available in PMC 2023 Apr 1. (PMC7614323; doi:10.1088/1741-2552/acbc4a)
Supplement: Supplementary information [file EMS171841-supplement-Supplementary_information.pdf]

Supplementary information

A Phase locking value

The PLV is calculated in the Kuramoto model as

$$\text{PLV } p{:}1 = \frac{1}{N}\left|\sum_{k=1}^{N} e^{i\psi(t_k)}\right|, \tag{S.1}$$

and quantifies the phase concentration of the order parameter at the time of stimulation, considering all stimulation pulses. PLV p:1 will therefore detect p:1 entrainment, for any $p \geq 1$. We also consider

$$\text{PLV } p{:}2 = \frac{1}{\lfloor N/2 \rfloor}\left|\sum_{k=1}^{\lfloor N/2 \rfloor} e^{i\psi(t_{2k})}\right|, \tag{S.2}$$

with $\lfloor . \rfloor$ the floor function. PLV p:2 only considers every other stimulation pulse, and will detect p:2 entrainment ($p \geq 1$), which includes for example 1:2 and 3:2 entrainment, but also 1:1 and 2:1 entrainment. Thus, to only detect $(2p-1)$:2 entrainment (in particular the 1:2 and 3:2 tongues), we introduce PLV $(2p-1)$:2 = PLV p:2 − PLV p:1.

B Simulating neural oscillators coupled through Hodgkin-Huxley electrotonic coupling

To verify that dithered stimulation is still effective in populations of neural oscillators coupled through electrotonic coupling derived from the Hodgkin-Huxley model [51], we also considered a modified version of equation (11) in the main text. In this modified model, the time evolution of the phase φ_k of the kth oscillator is described by

$$\mathrm{d}\varphi_k = \left[\omega_k + \frac{\kappa}{M}\sum_{l=1}^{M} G_e(\varphi_k - \varphi_l) + I(t)Z(\varphi_k)\right]\mathrm{d}t + \xi \mathrm{d}W_k, \tag{S.3}$$

where G_e denotes the electrotonic coupling function derived from the Hodgkin-Huxley model in [51] (see Fig S.9).

To get a large 2:1 synchronisation region in the absence of dithering for meaningful testing, we chose $\kappa = 300$, $\xi = 7.9$, and sampled the ω_k's from a Lorentzian distribution centered on the frequency considered ($f_0/[2\pi]$) and of width 20 Hz.

C Frequencies used in toggling analysis

The frequencies used in the first two rows of Fig 6 in the main text and Fig S.6 are

$$\{120, 130, 141.8\} \text{ Hz.}$$

The frequencies used in rows three and four of Fig 6 in the main text and Fig S.6 are

$$\{100, 108.33, 118.18, 130, 144.44, 162.50, 185.71\} \text{ Hz.}$$

The frequencies used in the last two rows of Fig 6 in the main text, and Figs S.6 and S.7 are

$$\{100, 104, 108.33, 113.04, 118.18, 123.81, 130, 136.84, 144.44, 152.94, 162.50, 173.33, 185.71\} \text{ Hz.}$$

D Supplementary figures

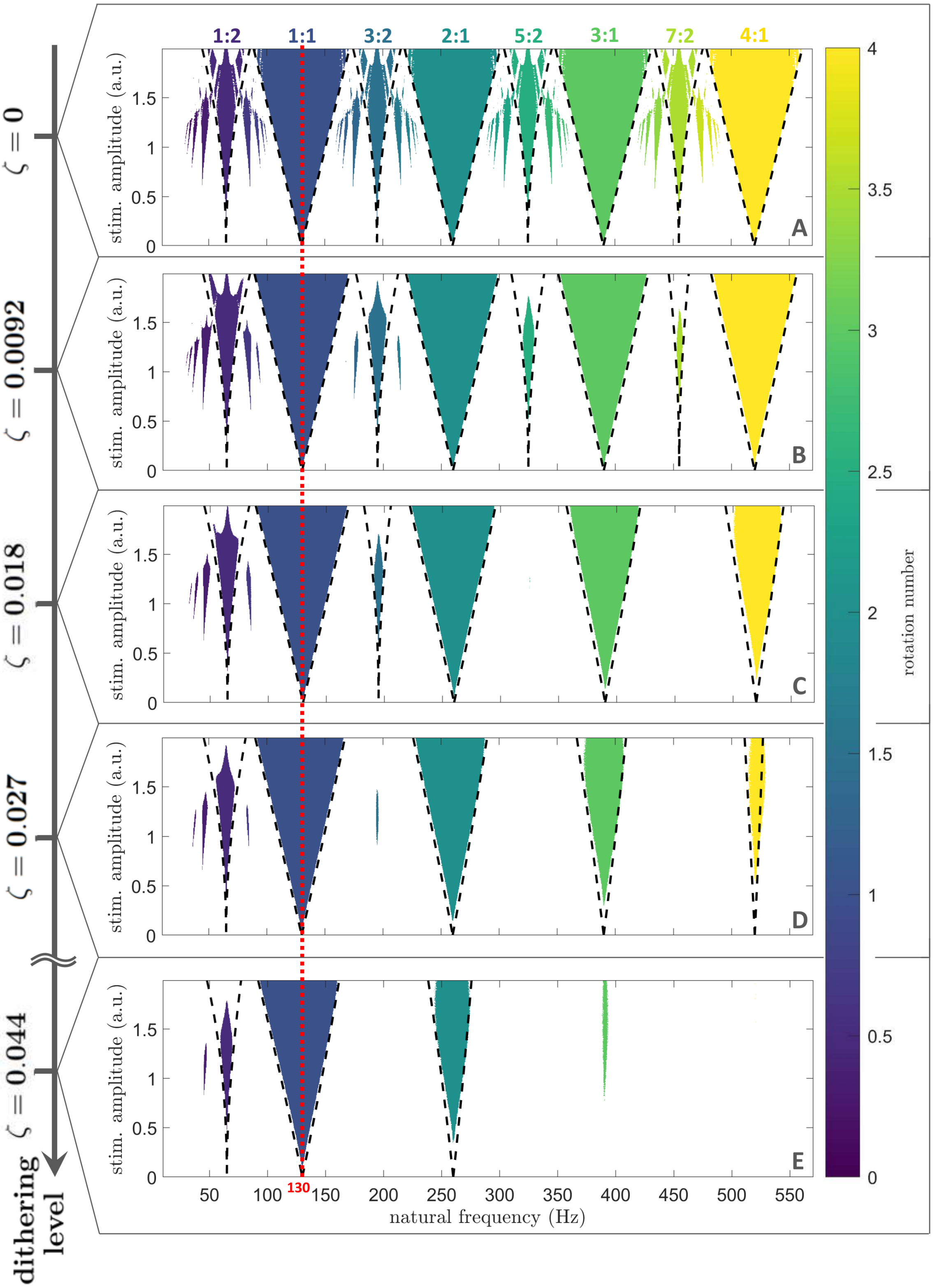

Figure S.1: p:1 and $(2p-1)$:2 Arnold tongues with p up to 4: validation of theoretical results and the efficacy of dithered stimulation. Frequency locking regions in the oscillator frequency/stimulation amplitude space. Only regions of frequency-locking (determined as presented in Section 4.2 in the main text), are shown in color. The color scale represents the rotation number. Dithering level increases from top to bottom, and theoretical tongue boundaries (equations (6) and (9)) are shown by black dashed lines. The frequency corresponding to the mean stimulation period is indicated by a red dashed line. An animation with finer steps in dithering levels is provided as supplementary material (jneacbc4asupp3.mp4, see Video S.2 for caption). For each natural frequency, stimulation amplitude, and dithering value, the rotation number is averaged over 10 repeats, with 10^4 stimulation pulses per repeat. Higher dithering levels, resulting in only the 1:1 tongue being stable, are shown in Fig 3A in the main text.

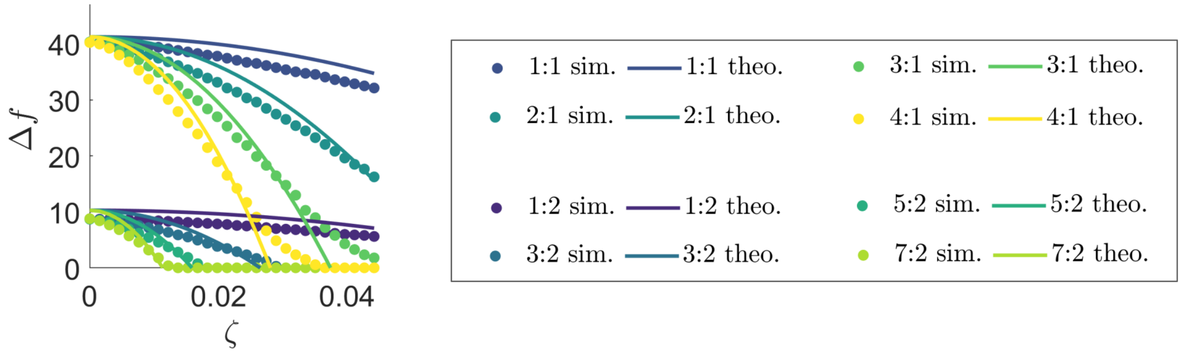

Figure S.2: Width of p:1 and $(2p-1)$:2 Arnold tongues with p up to 4: comparison of simulations and theoretical results. Comparing tongue width obtained from equations (7)/(10) and simulations, as a function of the dithering level, for $I = 1$. For each natural frequency and dithering value, the rotation number from simulations is averaged over 10 repeats, with 10^4 stimulation pulses per repeat. Higher dithering levels, resulting in only the 1:1 tongue being stable, are shown in Fig 3C2 in the main text.

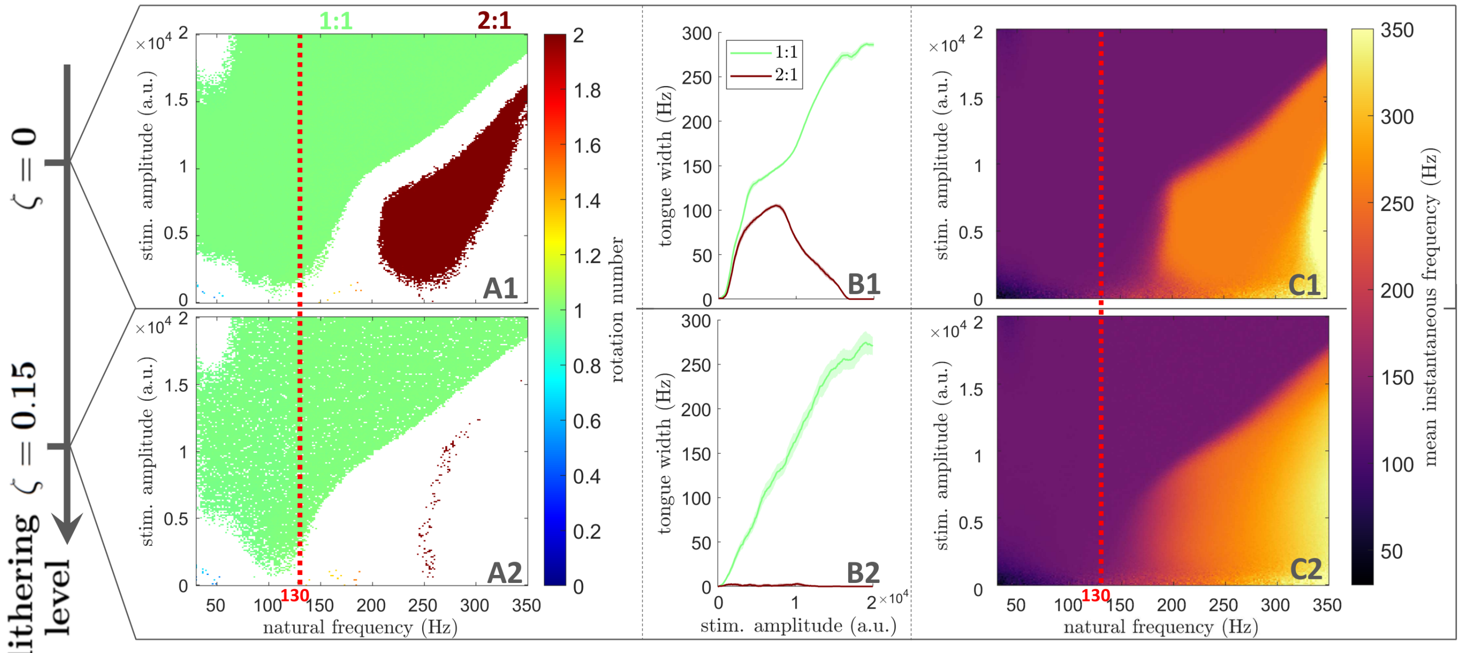

Figure S.3: Arnold tongues disappear at lower dithering levels for higher-order entrainment than for 1:1 entrainment in populations of neural oscillators coupled using Hodgkin-Huxley electrotonic coupling. Dithering level (ζ) increases from the top row to the bottom row. **A:** Frequency locking regions in the natural frequency/stimulation amplitude space. Only regions of frequency-locking (determined as presented in Section 4.2 in the main text), are shown in color. The color scale represents the rotation number. **B:** Arnold tongue width as a function of stimulation amplitude. **C:** Mean instantaneous frequency (represented by the color scale) in the natural frequency/stimulation amplitude space. In **A** and **C**, for each natural frequency, stimulation amplitude, and dithering value, the rotation number or mean instantaneous frequency are averaged over 5 repeats, with 400 stimulation pulses per repeat. The frequency corresponding to the mean stimulation period is indicated by a red dashed line. The modified model used in this figure is defined in Section B.

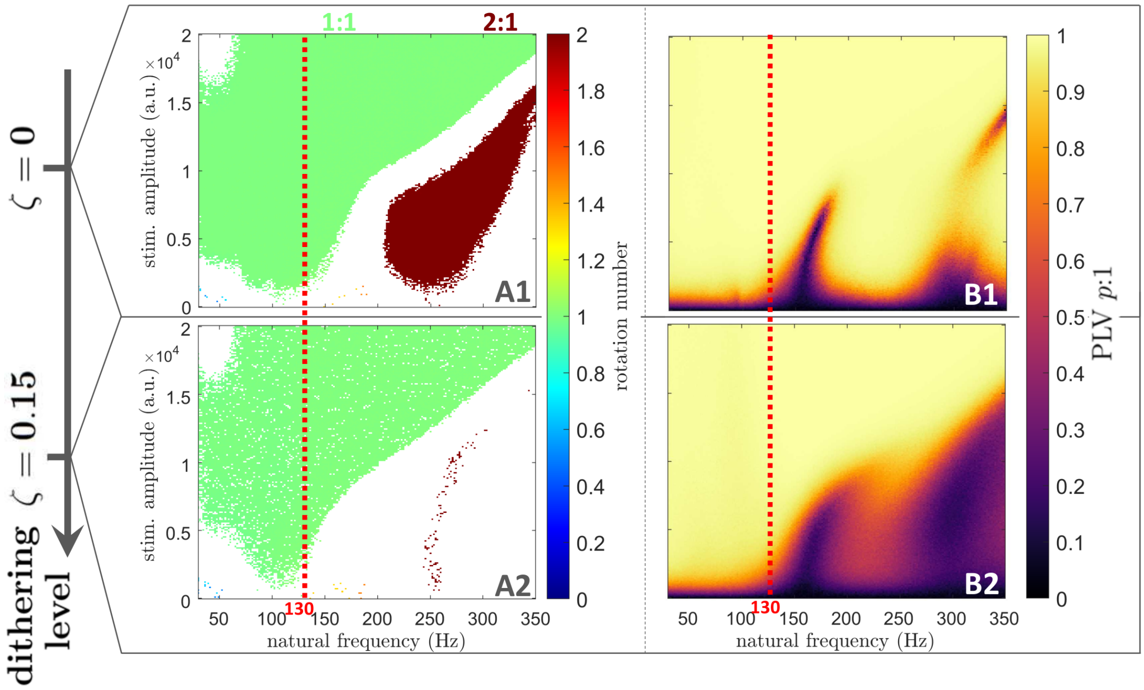

Figure S.4: The efficacy of dithered stimulation is confirmed by PLV analysis in populations of neural oscillators coupled using Hodgkin-Huxley electrotonic coupling. Dithering level (ζ) increases from the top row to the bottom row. **A:** Frequency locking regions in the natural frequency/stimulation amplitude space. Only regions of frequency-locking (determined as presented in Section 4.2 in the main text), are shown in color. The color scale represents the rotation number. **B:** PLV p:1 (color scale) in the natural frequency/stimulation amplitude space. Here, PLV p:1 detects 1:1 and 2:1 entrainment. In all panels, for each natural frequency, stimulation amplitude, and dithering value, the rotation number or mean instantaneous frequency is averaged over 5 repeats, with 400 stimulation pulses per repeat. The frequency corresponding to the mean stimulation period is indicated by a red dashed line. For $\zeta = 0.15$, 2:1 entrainment has disappeared while 1:1 entrainment is still supported (compare **B2** to **B1**). See Section A for more details on the PLV. The modified model used in this figure is defined in Section B.

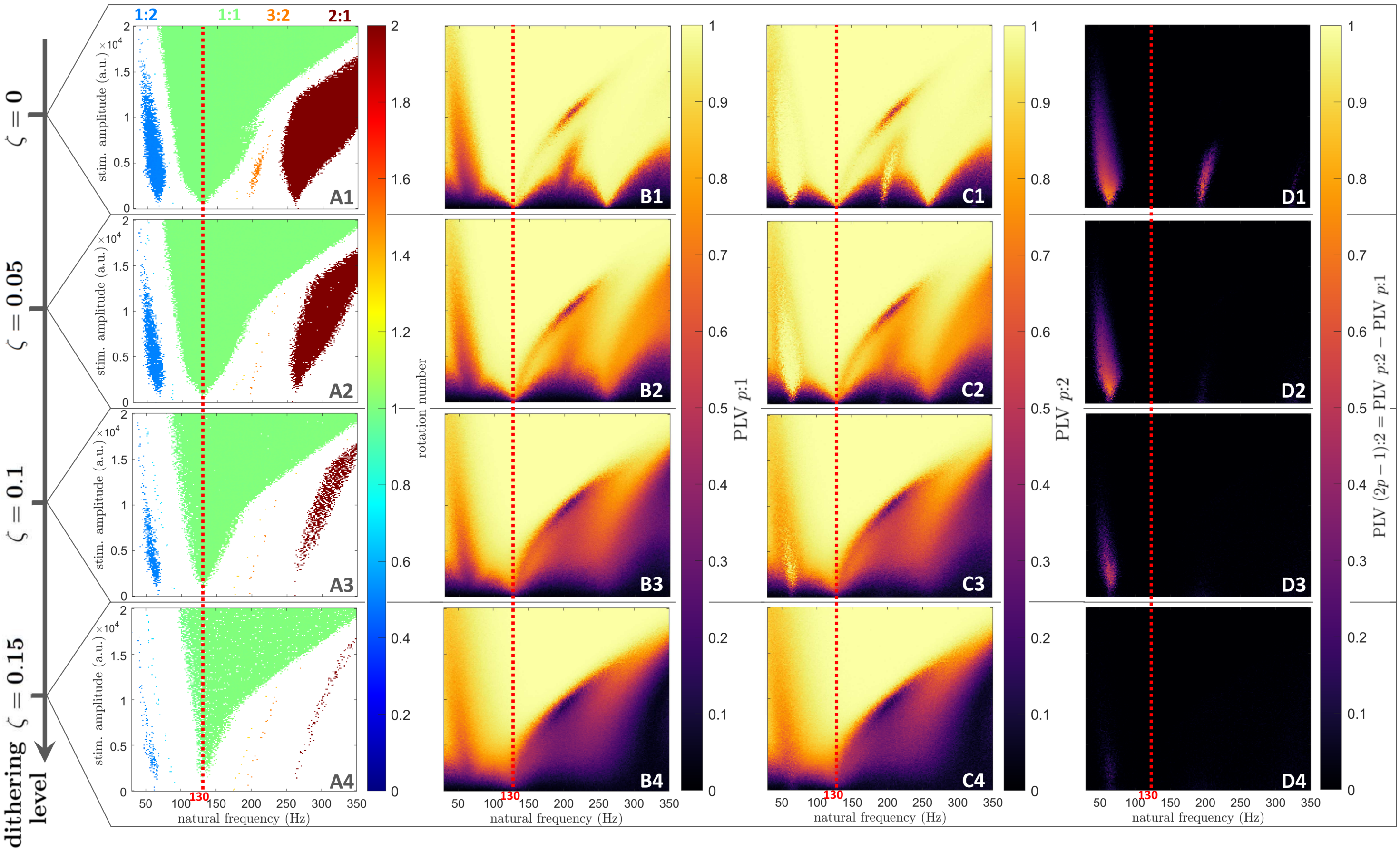

Figure S.5: The efficacy of dithered stimulation is confirmed by PLV analysis in populations of coupled neural oscillators. Dithering level (ζ) increases from the top row to the bottom row. **A:** Frequency locking regions in the natural frequency/stimulation amplitude space. Only regions of frequency-locking (determined as presented in Section 4.2 in the main text), are shown in color. The color scale represents the rotation number. **B:** PLV p:1 (color scale) in the natural frequency/stimulation amplitude space. Here, PLV p:1 detects 1:1 and 2:1 entrainment. **C:** PLV p:2 (color scale) in the natural frequency/stimulation amplitude space. Here, PLV p:2 detects 1:2 and 3:2 entrainment, but also 1:1 and 2:1 entrainment. **D:** PLV $(2p-1)$:2 (color scale) in the natural frequency/stimulation amplitude space, obtained as PLV p:2 - PLV p:1. Here, PLV $(2p-1)$:2 detects 1:2 and 3:2 entrainment. In all panels, for each natural frequency, stimulation amplitude, and dithering value, the rotation number or mean instantaneous frequency is averaged over 5 repeats, with 400 stimulation pulses per repeat. The frequency corresponding to the mean stimulation period is indicated by a red dashed line. For $\zeta = 0.15$, 2:1 entrainment has disappeared while 1:1 entrainment is still supported (compare **B4** to **B1**). 1:2 and 3:2 entrainment have also disappeared (compare **D4** to **D1**). See Section A for more details on the PLV.

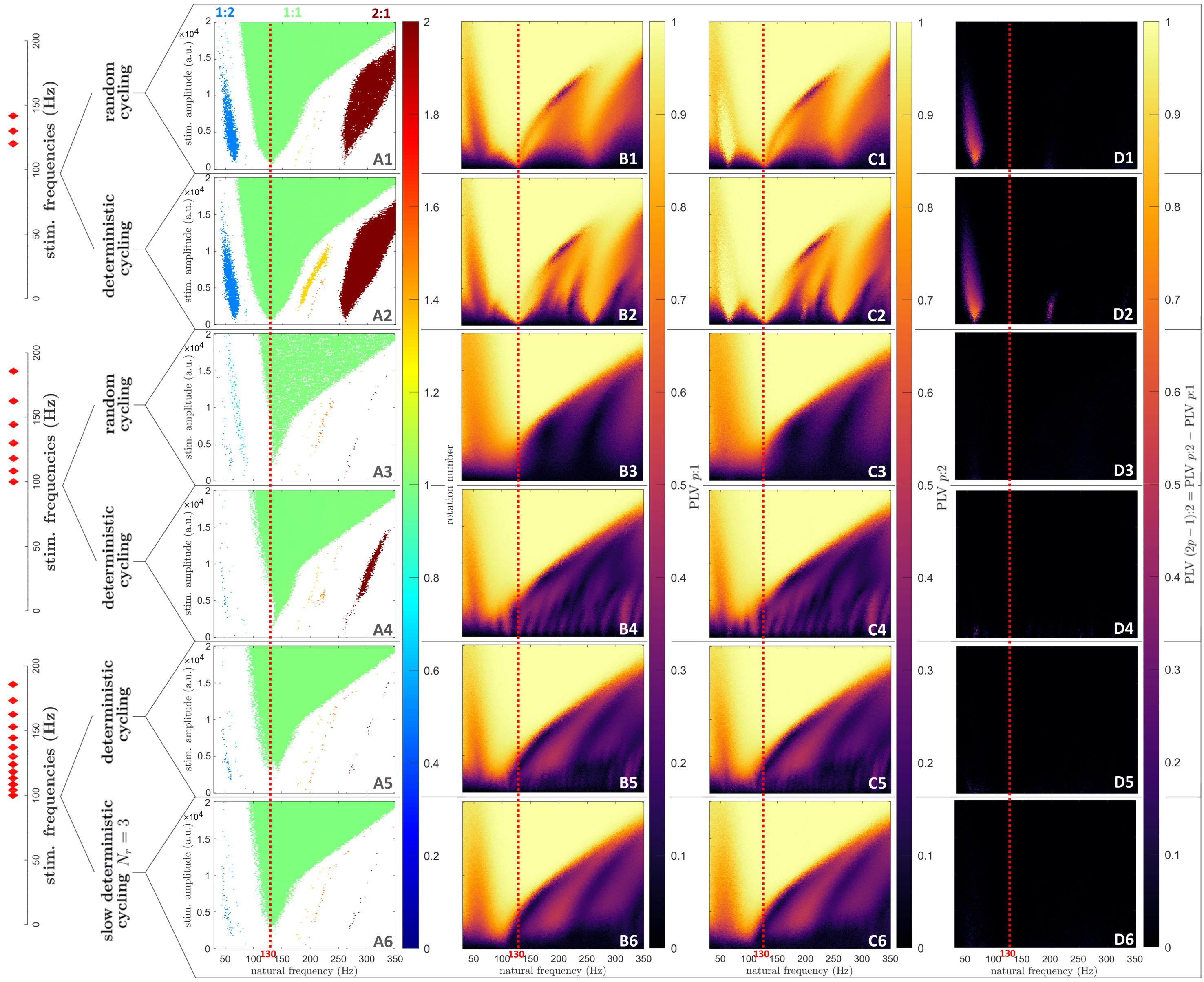

Figure S.6: The efficacy of the stimulation frequency toggling approach is confirmed by PLV analysis in populations of coupled neural oscillators. Each row correspond to a particular type of pulse train, as indicated on the left of the figure. For slow deterministic cycling (last row), $N_r = 3$. **A:** Frequency locking regions in the natural frequency/stimulation amplitude space. Only regions of frequency-locking (determined as presented in Section 4.2 in the main text), are shown in color. The color scale represents the rotation number. **B:** PLV p:1 (color scale) in the natural frequency/stimulation amplitude space. Here, PLV p:1 detects 1:1 and 2:1 entrainment. **C:** PLV p:2 (color scale) in the natural frequency/stimulation amplitude space. Here, PLV p:2 detects 1:2 and 3:2 entrainment, but also 1:1 and 2:1 entrainment. **D:** PLV $(2p-1)$:2 (color scale) in the natural frequency/stimulation amplitude space, obtained as PLV p:2 - PLV p:1. Here, PLV $(2p-1)$:2 detects 1:2 and 3:2 entrainment. In all panels, for each natural frequency, stimulation amplitude, and dithering value, the rotation number or mean instantaneous frequency is averaged over 5 repeats, with 400 stimulation pulses per repeat. The frequency corresponding to the mean stimulation period is indicated by a red dashed line. The stimulation frequencies used are plotted as red diamonds on the left of the figure (numerical values are given in Section C). See Section A for more details on the PLV.

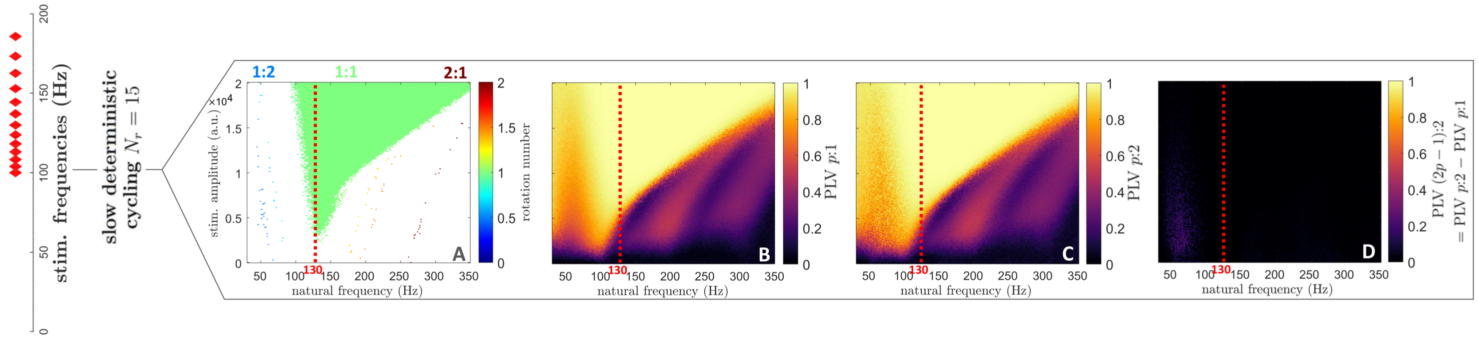

Figure S.7: Upper limit for N_r in the stimulation frequency toggling approach – PLV analysis in populations of coupled neural oscillators. The pulse train is detailed on the left of the figure (slow deterministic cycling, $N_r = 15$). **A:** Frequency locking regions in the natural frequency/stimulation amplitude space. Only regions of frequency-locking (determined as presented in Section 4.2 in the main text), are shown in color. The color scale represents the rotation number. **B:** PLV p:1 (color scale) in the natural frequency/stimulation amplitude space. Here, PLV p:1 detects 1:1 and 2:1 entrainment. **C:** PLV p:2 (color scale) in the natural frequency/stimulation amplitude space. Here, PLV p:2 detects 1:2 and 3:2 entrainment, but also 1:1 and 2:1 entrainment. **D:** PLV $(2p-1)$:2 (color scale) in the natural frequency/stimulation amplitude space, obtained as PLV p:2 - PLV p:1. Here, PLV $(2p-1)$:2 detects 1:2 and 3:2 entrainment. In all panels, for each natural frequency, stimulation amplitude, and dithering value, the rotation number or mean instantaneous frequency is averaged over 5 repeats, with 400 stimulation pulses per repeat. The frequency corresponding to the mean stimulation period is indicated by a red dashed line. The stimulation frequencies used are plotted as red diamonds on the left of the figure (numerical values are given in Section C). See Section A for more details on the PLV.

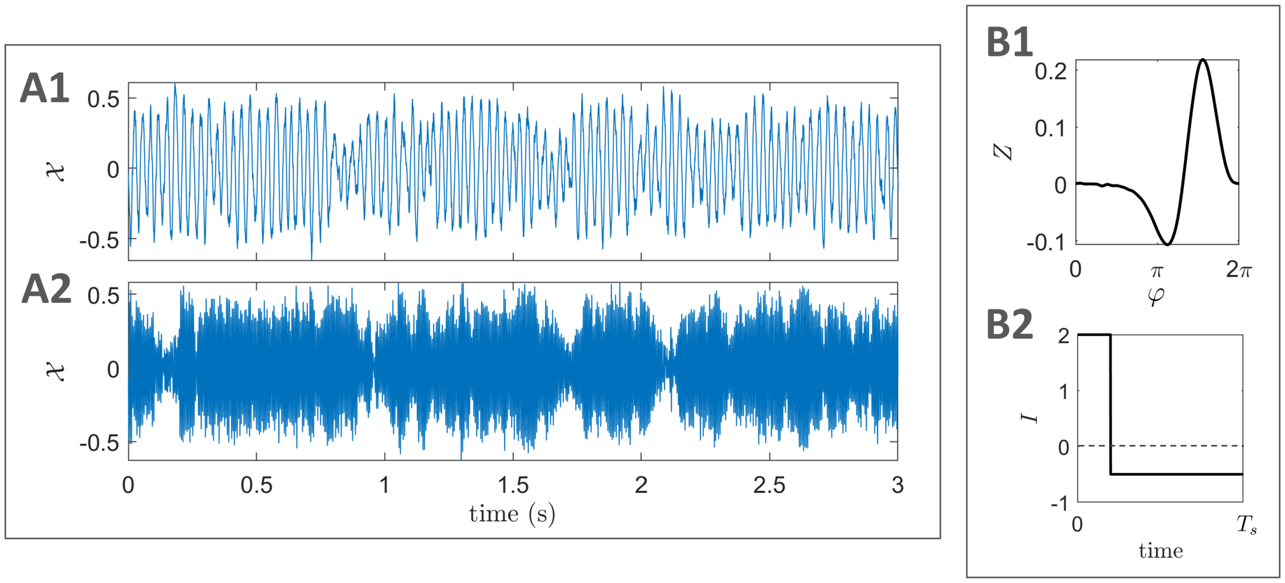

Figure S.8: Kuramoto model outputs in the absence of stimulation; PRC and stimulation pulse. Example outputs from the Kuramoto model for the chosen parameters in the absence of stimulation for $f_0 = 30$ Hz **(A1)** and $f_0 = 185$ Hz **(A2)**. The PRC of the oscillators is taken from the standard Hodgkin-Huxley neuron model **(B1)**. The charge-balanced rectangular stimulation pulse used in simulations is shown in **B2** (T_s is the stimulation period). The pulse shown has an energy of 1 a.u. over T_s, and is taken to be the base pulse for a stimulation amplitude of 1 a.u. in simulations.

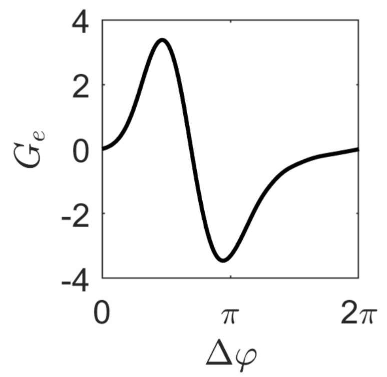

Figure S.9: Electrotonic coupling function for the Hodgkin-Huxley equations (derived in [51]). The electrotonic coupling function is plotted as a function of the phase difference $\Delta\varphi$.

E Captions of videos

Video S.1: Animation showing that Arnold tongues disappear at lower dithering levels for higher order entrainment than for 1:1 entrainment in uncoupled neural oscillators (jneacbc4asupp2.mp4). **Top:** Frequency locking regions in the oscillator frequency/stimulation amplitude space. Only regions of frequency-locking (determined as presented in Section 4.2 in the main text), are shown in color. The color scale represents the rotation number. The dithering level (ζ) increases with time, and theoretical tongue boundaries (equations (6) and (9)) are shown by black dashed lines. For each natural frequency, stimulation amplitude, and dithering value, the rotation number is averaged over 10 repeats, with 10^4 stimulation pulses per repeat. **Bottom:** Example train of stimulation triggers for the given dithering level.

Video S.2: Animation validating theoretical results and the efficacy of dithered stimulation for p:1 and $(2p-1)$:2 Arnold tongues with p up to 4 (jneacbc4asupp3.mp4). Top: Frequency locking regions in the oscillator frequency/stimulation amplitude space. Only regions of frequency-locking (determined as presented in Section 4.2 in the main text), are shown in color. The color scale represents the rotation number. The dithering level (ζ) increases with time, and theoretical tongue boundaries (equations (6) and (9)) are shown by black dashed lines. For each natural frequency, stimulation amplitude, and dithering value, the rotation number is averaged over 10 repeats, with 10^4 stimulation pulses per repeat. Higher dithering levels, resulting in only the 1:1 tongue being stable, are shown in Video S.1. **Bottom:** Example train of stimulation triggers for the given dithering level.
